# Optimal Sacrificial Domains in Mechanical Polyproteins: *S. epidermidis* Adhesins Are Tuned for Work Dissipation

**DOI:** 10.1021/jacsau.2c00121

**Published:** 2022-05-18

**Authors:** Haipei Liu, Zhaowei Liu, Byeongseon Yang, Joanan Lopez Morales, Michael A. Nash

**Affiliations:** † Department of Chemistry, University of Basel, 4058 Basel, Switzerland; ‡ Department of Biosystems Science and Engineering, ETH Zurich, 4058 Basel, Switzerland

**Keywords:** AFM, single-molecule force spectroscopy, bacterial adhesion, sacrificial domains, protein engineering, biophysics

## Abstract

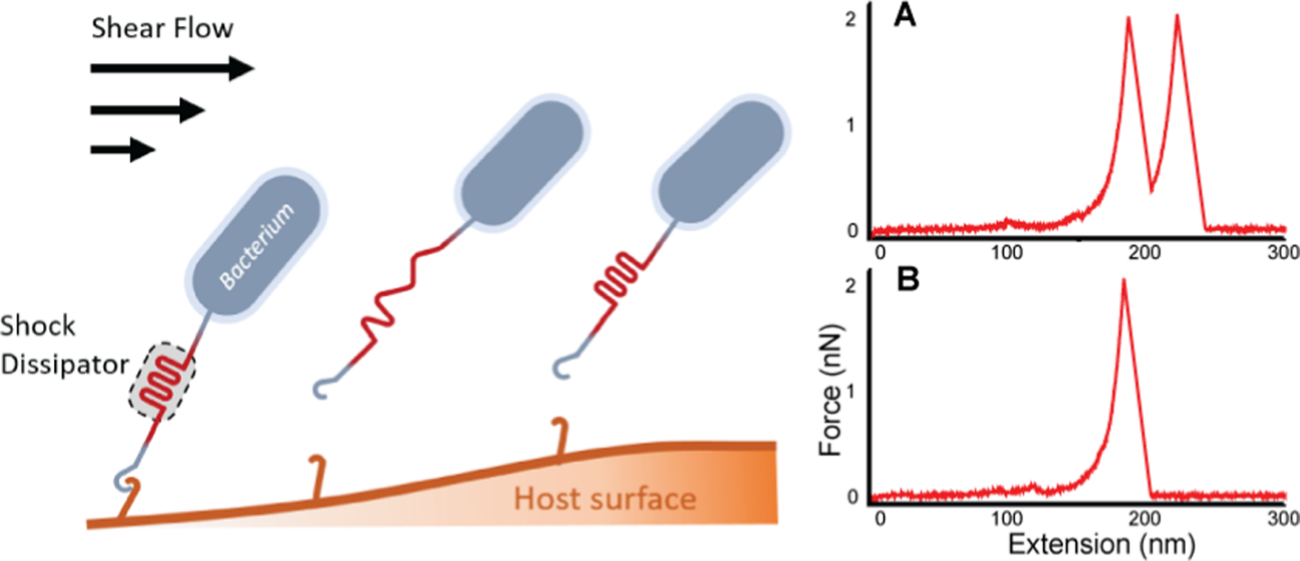

The opportunistic pathogen *Staphylococcus epidermidis* utilizes a multidomain surface adhesin protein to bind host components and adhere to tissues. While it is known that the interaction between the SdrG receptor and its fibrinopeptide target (FgB) is exceptionally mechanostable (∼2 nN), the influence of downstream B domains (B1 and B2) is unclear. Here, we studied the mechanical relationships between folded B domains and the SdrG receptor bound to FgB. We used protein engineering, single-molecule force spectroscopy (SMFS) with an atomic force microscope (AFM), and Monte Carlo simulations to understand how the mechanical properties of folded sacrificial domains, in general, can be optimally tuned to match the stability of a receptor–ligand complex. Analogous to macroscopic suspension systems, sacrificial shock absorber domains should neither be too weak nor too strong to optimally dissipate mechanical energy. We built artificial molecular shock absorber systems based on the nanobody (VHH) scaffold and studied the competition between domain unfolding and receptor unbinding. We quantitatively determined the optimal stability of shock absorbers that maximizes work dissipation on average for a given receptor and found that natural sacrificial domains from pathogenic *S. epidermidis* and *Clostridium perfringens* adhesins exhibit stabilities at or near this optimum within a specific range of loading rates. These findings demonstrate how tuning the stability of sacrificial domains in adhesive polyproteins can be used to maximize mechanical work dissipation and serve as an adhesion strategy by bacteria.

## Introduction

*Staphylococcus epidermidis* is a common commensal bacterium of the skin and nasal microbiome that colonizes implanted medical devices and causes infection. Although *S. epidermidis* possesses a smaller number of virulence factors than *Staphylococcus aureus*, it maintains a number of cell-wall anchored adhesins that promote biofilm formation and host infection by binding to extracellular matrix and blood proteins including collagen, fibronectin, and fibrinogen.^1,2^ These blood and matrix components coat implanted medical devices as part of the foreign body response and serve as potential reservoirs for infection. This has led to a need for understanding bacterial adhesion mechanisms in an effort to combat antibiotic-resistant infections.

Among the various cell-wall-anchored adhesins of *S. epidermidis*, SdrG is a member of the microbial surface components recognizing adhesive matrix molecules (MSCRAMMs) family. SdrG is a multidomain polyprotein that has received significant interest due to the ultrastable force-activated interaction it forms with the N-terminal fibrinopeptide of the fibrinogen Bβ-chain (FgB)^3,4^ that requires >2 nN to dissociate at 10^5^ pN/s but maintains moderate thermodynamic affinity with *K*_D_ = ∼400 nM.^5^ Adjacent to the SdrG N2-N3 receptor domains (referred to as the A region) are two globular B domains (B1 and B2) with ultrastable mechanical properties typical of Gram-positive adhesins,^6,7^ requiring >2 nN of tension to unfold at 10^5^ pN/s.^8^ These mechanostable adhesin proteins are known to be critical for maintaining tissue adhesion under flow and contributing to biofilm formation on medical device surfaces.^9^

Here, we studied the loading response of engineered polyproteins mimicking those found in the *S. epidermidis* native receptor, comprising SdrG (N2-N3) fused with B1 and B2 domains. Specifically, we considered the amount of mechanical work that is required to unfold and stretch these polyproteins under constant speed and constant loading rate protocols. When force is applied to anchored B domains using the SdrG:FgB interaction as a pulling anchor (analogous to the case *in vivo*), the observed unfolding force distribution of the B domains is biased toward lower forces. This arises because the SdrG:FgB interaction used to apply tension across the B domains has finite stability and frequently breaks prior to B domain unfolding. The questions that arise from this scenario are: (1) Given this biasing effect, how can we measure the true mechanical unfolding parameters of B domains? (2) How does the stability of B domains influence the amount of mechanical work required to stretch the polyprotein through the SdrG:FgB interaction? (3) Is there optimal domain stability that maximizes mechanical work dissipation upon unfolding and stretching the sacrificial domains (on average), and how does that optimum compare with native B domain mechanics?

**Figure 1:**
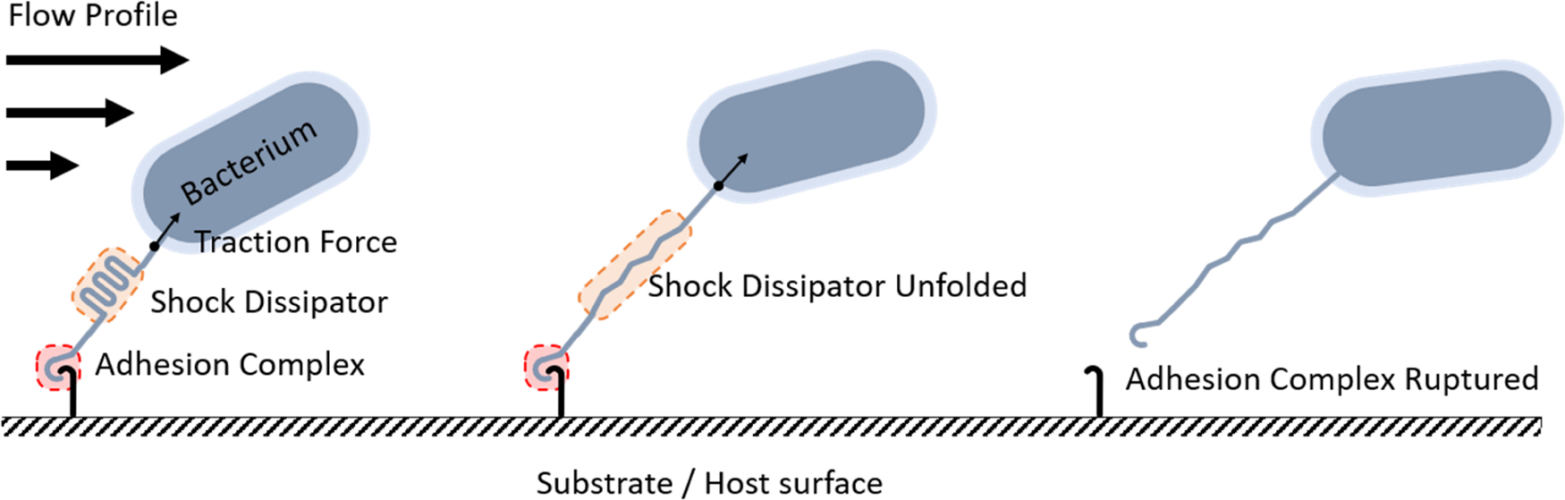
Work dissipation in adhesive polyproteins. A bacterium adheres to a substrate through an adhesion complex. A sacrificial shock dissipator domain with optimal mechanical properties buffers mechanical fluctuations and helps maintain the integrity of the surface adhesion complex under hydrodynamic forces.

Mechanical force can regulate protein structure and function in diverse ways.^10−12^ Protein unfolding releases hidden biopolymer contour length that requires the input of mechanical work to extend, and this effect can act as a shock dissipator in biomaterials under tension (Figure 1).^13−15^ This concept of sacrificial bonds in biomaterials is well established, with strengthening effects attributed to structural changes at the protein level (e.g., unfolding) in diverse systems including bone,^16−18^ muscle,^19,20^ fibrin,^21,22^ collagen,^23^ as well as in synthetic materials.^24,25^ Here, we analyze theoretical and practical underpinnings of this behavior. We generalize the problem of mechanical work dissipation in polyproteins by considering the unfolding response of fingerprint (FP) domains (i.e., independently foldable globular domains embedded in polyproteins) using receptor–ligand (RL) complexes as pulling anchors. Building on our previous Monte Carlo analysis,^26^ we confirm the FP biasing effect experimentally and demonstrate how quantification of an experimentally observable parameter (eta, η), representing the fraction of unbinding trajectories that exhibit FP unfolding, can be used in a correction algorithm on biased experimental data to recover the true unbiased energy landscape parameters of FP domains. We apply this formalism to study the mechanical properties of native adhesive polyproteins, focusing on the FgB:SdrG-B1-B2 system from *S. epidermidis* and the FIVAR-Dockerin:Cohesin system from *Clostridium perfringens*.

We first built artificial polyprotein systems using the nanobody (i.e., single-domain VHH antibody) scaffold fused with FP domains and validated our correction algorithm by comparison to unbiased FP unfolding data obtained with high-stability pulling handles. We then applied our correction algorithm to simulated data as well as experimental data from the SdrG:FgB system to correct the biased unfolding energy landscape parameters for the B2 domain. Finally, we considered the optimal FP domain stability that maximizes mechanical work dissipation for a given receptor and found a nonlinear behavior with a clear optimal value. This optimal FP stability represents a balance between strong FPs, which dissipate large amounts of work but frequently do not unfold prior to RL breakage, and weak FPs that dissipate smaller amounts of work upon unfolding and stretching but do so more frequently prior to RL breakage.

We show that for two pathogenic adhesive polyprotein systems (*S. epidermidis* and *C. perfringens* adhesins), the native FP domains exhibit stabilities at or near the optimal value for a specific range of loading rates. We argue that by incorporating FPs with mechanical stability tuned slightly below their respective adjacent RL complexes, natural adhesins such as the *S. epidermidis* SrdG-B1-B2 and *C. perfringens* FIVAR-Dockerin systems have evolved optimal sacrificial domains that can potentially serve as targets in the development of antiadhesion therapies to combat infection.

### Theoretical Framework

The process of FP domain unfolding or RL complex rupture can be described as thermally driven escape over an energy barrier accelerated under external force, with a probability distribution of unfolding or rupture forces described by eq 1(27)
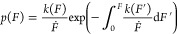
1where *Ḟ* is the loading rate and *k*(*F*) is the force-dependent off-rate. *k*(*F*) can take different functional forms, and most classically is given by the Bell–Evans model^28,29^
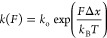
2with a distribution of first-passage forces given by
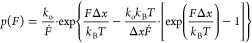
3where *k*_o_ and Δ*x* are the zero-force off-rate and position of the energy barrier, respectively; *k*_B_ is the Boltzmann constant; and *T* is the temperature. Assuming the Bell–Evans^28,29^ expression for the force-dependent off-rate (eq 2) and a constant loading rate, eq 1 can be solved analytically and used to fit experimentally measured unfolding or rupture force distributions in single-molecule force spectroscopy (SMFS) and extract energy landscape parameters, as shown in eq 3. In recent years, more sophisticated expressions for the force-dependent off-rate have been developed to account for shortening of the barrier position and rapid rebinding effects,^27,30−33^ and we note that these expressions for *k*(*F*) are also compatible with the analysis algorithm presented below.

**Figure 2:**
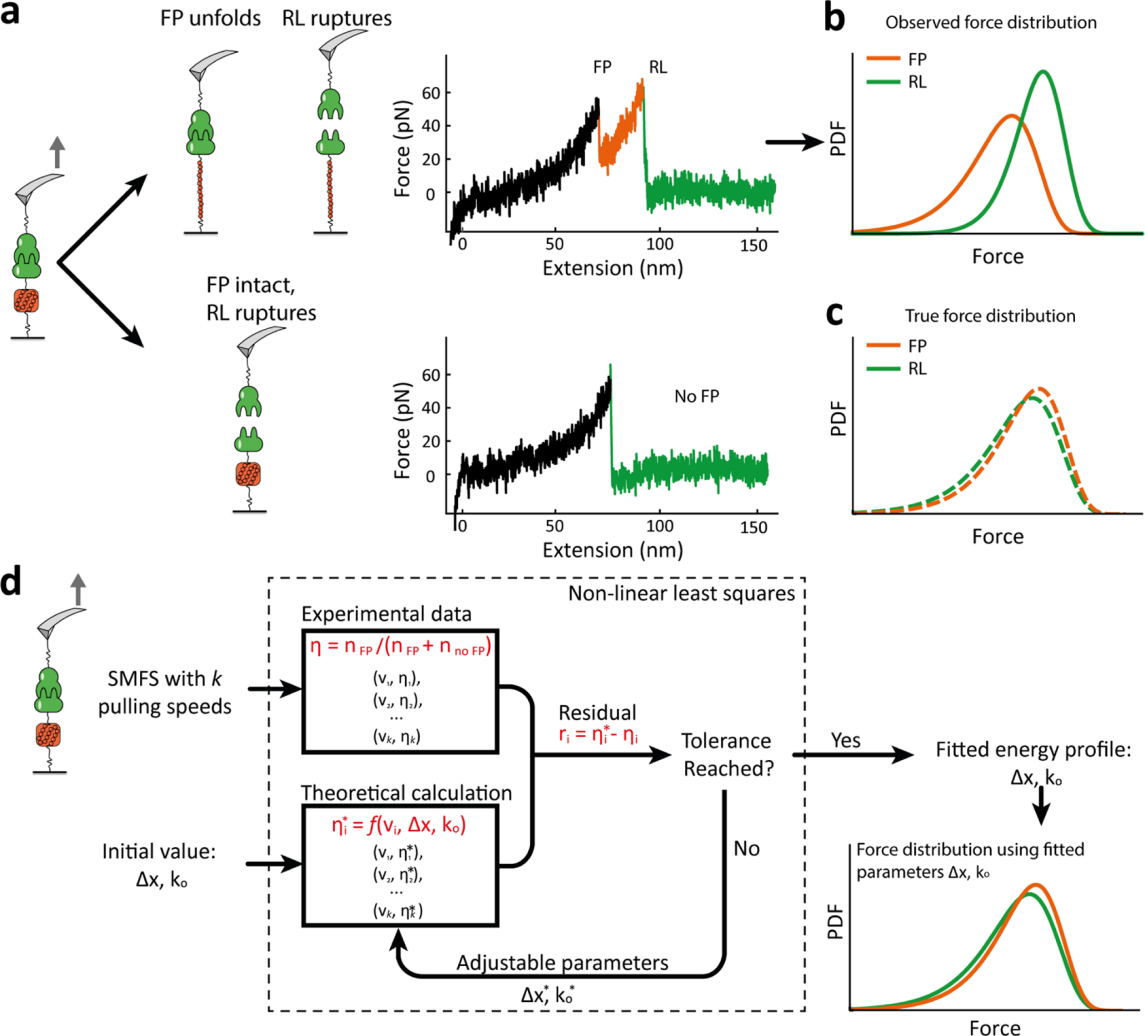
Biasing effect and correction algorithm based on η residuals. (a) Two pathways are possible in an atomic force microscope (AFM)-SMFS assay using an RL complex to unfold an FP domain. Typical experimental data (middle) showing FP unfolding followed by complex rupture (upper trace) or complex rupture prior to FP unfolding (lower trace). (b) Biased force distributions of RL rupture (green) and FP unfolding (orange) obtained from analysis of force extension exhibiting FP unfolding. (c) True force distribution (unbiased) of the RL rupture events can be obtained by analyzing traces from both pathways. To obtain the true distribution of FP unfolding forces, a correction algorithm is required. (d) Overview of the correction algorithm to extract the true distribution of FP unfolding forces from biased experimental AFM-SMFS observations using a nonlinear least-squares fitting of η. Initial guesses for energy landscape parameters (in this case, Bell–Evans *k*_0_ and Δ*x*) for the FP are obtained by direct fitting of the biased experimental FP unfolding force distribution. Based on the guess, the theoretical eta value (η*) is numerically computed using eqs 4 and 5 and compared with the experimentally observed η. This process is repeated with updated energy landscape parameters for the FP domain until the tolerance on η residuals is reached.

The biased unfolding force distribution for the FP domain, p*_FP_(*F*) (eq 4), is a continuous distribution modulated by the probability that the RL complex breaks at a force higher than that at which the FP unfolds. The biased distribution p*_FP_(*F*) is therefore proportional to the true FP unfolding force distribution *p*_FP_(*F*) multiplied by the cumulative probability that the RL complex ruptures at higher forces than the FP unfolding event and divided by a renormalization constant eta, η (eq 5), to define a probability density function. We previously showed^26^ that η represents the fraction of single-molecule trajectories exhibiting FP unfolding prior to RL rupture. It is straightforward to experimentally determine η by counting the number of force curves with and without FP unfolding events. Therefore, we hypothesized that η could be a good parameter on which to fit experimental SMFS results to correct for the biasing of the RL complex on the unfolding force distribution.
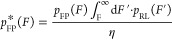
4
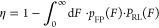
5
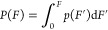
6It should be noted that in eqs 4–6 by which p*_FP_(*F*) is derived, all nonstar quantities in the expression equations represent the true force distributions. That is to say, p*_FP_(*F*) refers to the biased distribution of FP unfolding forces, *p*_FP_(*F*) refers to the unbiased distribution of FP unfolding forces, and *p*_RL_(*F*) refers to the unbiased distribution of RL rupture forces. We obtained the unbiased distribution of RL rupture forces by analyzing all RL rupture events from both curve classes shown (Figure 2a).

Typically, theoretical treatment of dynamic force spectroscopy is performed under the assumption of a constant loading rate;^33^ however, in many instances, a constant pulling speed protocol is easier to implement experimentally. In the constant-speed scenario, the loading rate *Ḟ* is nonconstant but rather a function of the force and contour length. As a result, eq 4 cannot be solved analytically. Nevertheless, given analytical expressions for *p*_FP_(*F*) and *p*_RL_(*F*), the value of η can be numerically computed from the integrals in eqs 5 and 6.

### Validation of Fingerprint Domain Biasing Effect on Simulated and Experimental Datasets

Here, we show direct experimental confirmation of the FP biasing effect and validate our correction algorithm based on the minimization of residuals on η (Figure 2). By measuring the unfolding force distribution of an FP domain using either a low-strength or a high-strength RL complex as the pulling handle, we were able to generate datasets containing both biased and true FP unfolding force distributions, respectively. We then implemented our correction algorithm on the biased dataset to obtain corrected energy landscape parameters consistent with the observed η values. We also demonstrate the correction algorithm on purely synthetic (simulated) data.

To build a test system, we designed artificial polyproteins containing RL complexes and FP domains with a significant degree of overlap in their unfolding and rupture force probability distributions. As one FP domain, we chose the FIVAR (found in various architectures) domain from a *C. perfringens* toxin complex. This domain has been used as a low-force FP domain in a previous SMFS-AFM study,^34^ where it showed a single-step energy barrier requiring ∼50 pN to unfold at 10^3^–10^4^ pN/s. As a second FP domain, we used the 4th *F*-actin cross-linker filamin domain from *Dictyostelium discoideum* (ddFLN4),^35^ containing a low-force intermediate state along its unfolding pathway. For the high-strength RL complex, we used the SdrG:FgB interaction, and for the low-strength RL complex, we selected a single-domain camelid antibody (i.e., VHH nanobody) domain that forms a complex with the fluorescent protein mCherry. The VHH:mCherry low-strength RL complex ruptures also at ∼60 pN at 10^3^–10^4^ pN/s^34^ and exhibits a rupture force distribution with significant overlap with the FIVAR and ddFLN4 unfolding force distributions. We hypothesized that the weakness of the VHH:mCherry interaction would lead to a strongly biased unfolding force distribution for FIVAR and ddFLN4 and provide a good system on which to validate our correction algorithm.

**Figure 3:**
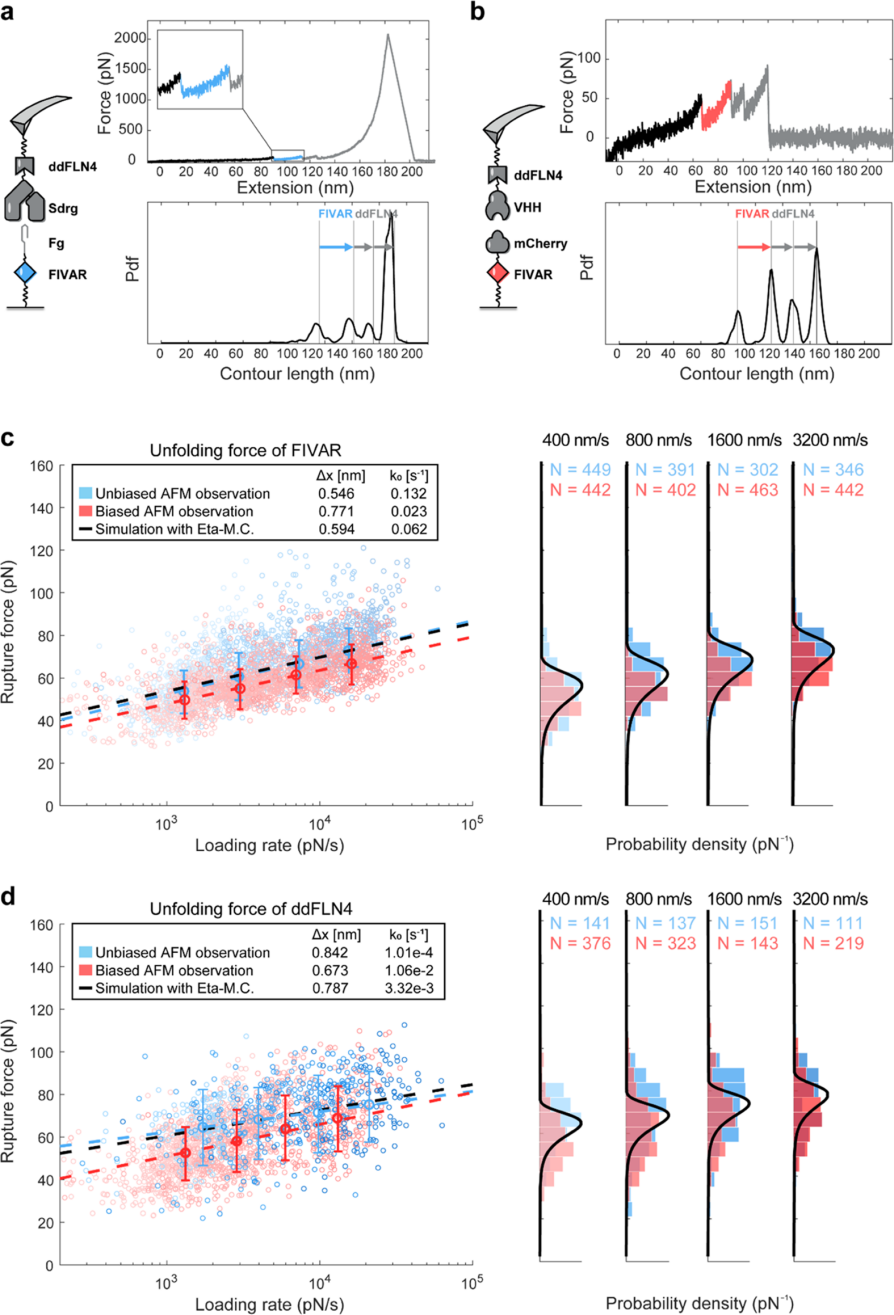
Experimental validation of biasing of ddFLN4 and FIVAR unfolding forces by the VHH:mCherry complex and implementation of correction algorithm. (a, b) AFM-SMFS measurements on FIVAR domain with (a) Sdrg:Fg complex (unbiased system) and (b) VHH:mCherry complex (biased system). Experimental AFM setup, representative force trace, and the aligned contour length histogram are shown. The unfolding of FIVAR domain with an ∼31 nm increment, followed by the two-step unfolding of the ddFLN4 FP domain with ∼35 nm increments could be identified from the contour length histogram. (c) Dynamic force spectrum of FIVAR unfolding forces obtained from both (a) unbiased system using SdrG:Fg complex (blue) and (b) biased system using VHH:mCherry complex (red). (d) Dynamic force spectrum of ddFLN4 unfolding forces obtained from both (a) unbiased system using SdrG:Fg complex (blue) and (b) biased system using VHH:mCherry complex (red). The most probable rupture force and loading rates were fit using the Bell–Evans model (dashed lines). Using the fitting approach based on minimizing η residuals, we obtained new energy landscape parameters corresponding to the black dashed line. In the right-hand-side plots of (c) and (d), the black solid line represents the distribution after algorithmic correction.

We cloned, recombinantly expressed, and purified multidomain polyproteins from *Escherichia coli*. Full amino acid sequences of the proteins used in these studies are reported in the Supporting Information. The constructs were: (i) SdrG-ddFLN4-His-ybbR; (ii) VHH-ddFLN4-His-ybbR; (iii) FgB-FIVAR-His-ybbR; and (iv) mCherry-FIVAR-His-ybbR, where His indicates a poly(6x) histidine tag for affinity chromatography purification and ybbR indicates the genetically encoded substrate for Sfp phosphopantetheinyl transferase^36^ for site-specific immobilization. Proteins were linked to cantilever or coverglass surfaces through ybbR tags.^37−39^ Large datasets consisting of tens of thousands of single-molecule AFM stretching and unfolding traces were acquired and screened for ddFLN4/FIVAR unfolding. We measured FIVAR unfolding and the first peak of ddFLN4 unfolding events observed in bound complexes between constructs (i:iii) and (ii:iv). These systems are identical in terms of the FPs but have different RL complexes used to apply tension across the FPs. Both systems were well behaved in AFM-SMFS assays, generating hundreds of usable SMFS traces exhibiting FP unfolding and RL rupture events, with contour length histograms that allowed domain assignment (Figure 3a,b) and quantification of the unfolding force distributions.

The biasing effect was clearly observed in the resulting FIVAR (Figure 3c) and ddFLN4 (Figure 3d) datasets. Due to the high (>2 nN) stability of the SdrG:FgB interaction, the measurement of FIVAR unfolding forces for samples (i:iii) was unbiased and represents the true distribution. When FIVAR was unfolded using VHH:mCherry as the pulling handle under configuration (ii:iv), the FIVAR unfolding force distribution was shifted downward to lower forces by on average 7.8% across the four pulling speeds from 400 to 3200 nm/s. When SdrG:FgB was used as the pulling handle (i:iii), all 1398 curves passing the ddFLN4 filter were found to have FIVAR unfolding events (η = 1). However, using VHH:mCherry only 45.1% of the total 3295 single-molecule force traces showed the FIVAR unfolding, indicating η = 0.451. FIVAR unfolding events that would have been observed at the upper end of the distribution were missed because VHH:mCherry broke and the tether between the cantilever and surface was lost prior to unfolding. Fitting of energy landscape parameters from the biased observations leads to errors and predicts probability density functions that are not consistent with the experimentally observed η; therefore, we developed a fitting algorithm based on minimizing the residuals of η (below) to correct for this effect.

### Correcting Energy Landscape Parameters by Minimizing η Residuals

**Figure 4:**
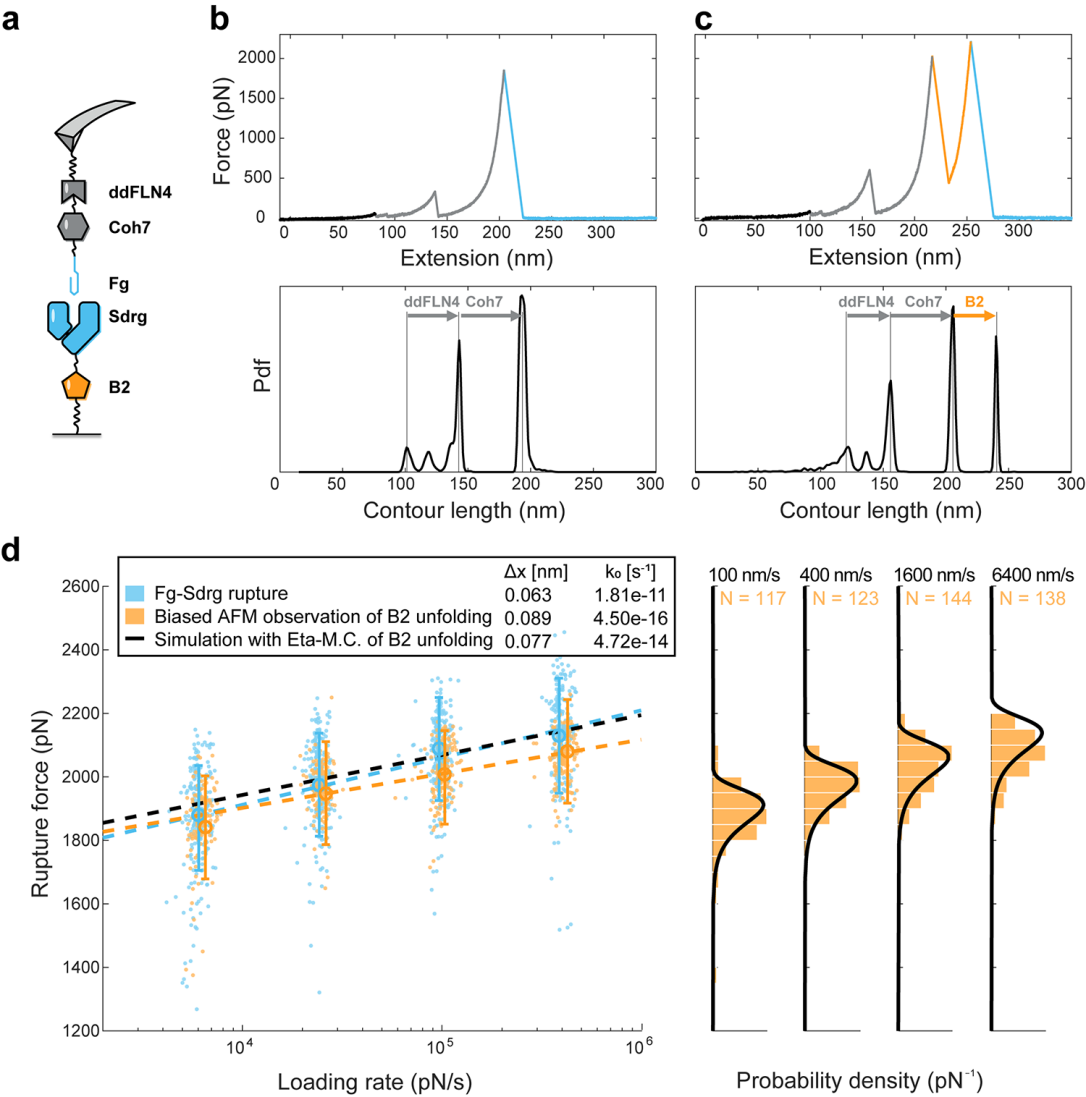
Extracting corrected B2 unfolding parameters from biased AFM-SMFS data. (a) AFM experimental setup. (b, c) Representative force traces and the aligned contour length histograms showing the two possible dissociation pathways for the SdrG-B2 system. (d) Dynamic force spectrum of the biased B2 unfolding force (orange) and Sdrg:FgB rupture events (blue). The most probable rupture forces and loading rates were fitted using the Bell–Evans model shown in dashed lines. We used the fitting approach based on minimizing residuals of η to obtain a corrected Bell–Evans expression for the loading rate dependency of B2 domain unfolding (black dashed line) and the corresponding unfolding force distributions (right, solid black lines).

The loading rate dependency of η for the VHH:mCherry (ii:iv) configuration was determined by the fraction of force curves showing FIVAR unfolding across a range of pulling speeds from 400 to 3200 nm/s. Using the biased FIVAR dataset as input, we obtained initial guesses for the energy landscape parameters for the Bell–Evans model with which we generated the closed-form Bell–Evans expression for the probability distribution of FIVAR unfolding forces (eq 3). The predicted η* value was then calculated by numerical integration following eqs 5 and 6. For the numerical integration step, the true energy landscape parameters of the RL complex are required. These were obtained by analyzing all RL rupture events regardless of the unfolding status of FP domains. The theoretically predicted η value thus obtained was higher than that observed experimentally because the stability of the FP was underestimated from the biased data. We next calculated the residuals between the theoretically predicted η* and the experimentally observed η as a function of loading rate. As shown in Figure 2D, nonlinear least-squares fitting was then used to iteratively update the guesses for the energy landscape parameters (*k*_o_ and Δ*x* for the Bell–Econtourvans model), generate a new closed-form expression for the Bell–Evans probability distribution function of FIVAR unfolding forces, and newly predict the expected η* values. Alternatively, the updated guesses could be passed into a numerical Monte Carlo simulation on the order of 10,000–100,000 force-extension curves to calculate the theoretical η*. This process was repeated until a close agreement between the theoretically predicted and experimentally observed η values was obtained. Depending on the force range and instrument sensitivity, the accuracy of this correcting strategy may be limited by experimental error, particularly for low-force systems (<50 pN). The correction approach was validated on experimental datasets (Figures 3 and 4) with corrected energy profiles given in Table 1. In addition, it was also tested on a synthetic dataset generated by Monte Carlo simulation (Supporting Information Figure 1).

**Table 1:** Parameter Estimates (±SE) Obtained by Fitting Biased Experimental Data and Applying the Corrections Algorithm to Minimize Residuals on η

	energy profile for FP unfolding					
	biased observation		unbiased observation		corrected	
FP	Δ*x*_biased_ [nm]	Ln (*k*_0,biased_)	Δ*x* [nm]	Ln (*k*_0_)	Δ*x* [nm]	Ln (*k*_0_)
FIVAR	0.77 ± 0.03	–3.8 ± 0.2	0.55 ± 0.03	–2.0 ± 0.3	0.59 ± 0.05	–2.7 ± 0.7
ddFLN4	0.67 ± 0.04	–4.5 ± 0.8	0.84 ± 0.08	–9.2 ± 1.7	0.79 ± 0.17	–5.7 ± 0.5
B2	0.089 ± 0.018	–35 ± 5			0.076 ± 0.012	–31 ± 3

### Biasing Effects in FgB:SdrG-B2 Complexes

In the prior section, we built an artificial system with designed overlaps in the distributions of FP unfolding forces and RL complex rupture events. The high-force SdrG:FgB complex was sufficiently strong to unfold both FIVAR and ddFLN4 in all traces, so we could compare the results obtained with the correction algorithm to the true distribution. However, when measuring very mechanostable FP domains such as B1 and B2 from SdrG adhesins, currently, no known molecular handle exists that is sufficiently stable to provide an unbiased analysis. Covalent bonding, with a rupture force of 1–2 nN under AFM setup,^40^ is not appropriate due to the lack of reversibility and lack of regeneration of bonding that is required for the AFM setup. Nonspecifically adsorbed polyproteins will detach typically below 1 nN. Therefore, for a subset of mechanostable FPs including B1 and B2, no direct unbiased measurement is possible. Here, we implement our correction algorithm on biased data from the B2 domain to obtain corrected energy landscape parameters describing its mechanical stability.

We produced the following proteins using genetic engineering and recombinant protein production in *E. coli*: (v) FgB-Coh7-ddFLN4-ELP-His-ybbR; (vi): SdrG(N1-N2)-B2-ELP-His-ybbR and site-specifically linked them to a glass surface and cantilever through the terminal ybbR tags. We performed AFM-SMFS measurements at pulling speeds of 100, 400, 1600, and 6400 nm/s and detected 813 specific rupture events. To ensure specificity of the single-molecule trajectories, two other FP domains (ddFLN4 and Coh7) were included that exhibit much lower unfolding forces (∼80 pN and ∼600 pN, respectively) than the SdrG:FgB complex. While ddFLN4 and Coh7 were unfolded in all of the obtained AFM traces (Figure 4a–c), the B2 domain was only observed in 64.2% of traces (η = 0.642), suggesting that the B2 distribution was biased toward lower forces. Fitting the B2 unfolding events with the Bell–Evans model using the biased AFM observation yielded the energy landscape parameters: (Δ*x* = 0.089 ± 0.018 nm, *k*_o_ = 4.5 × 10^–16^ ± 2.1 × 10^–15^ s^–1^). Using nonlinear least-squares fitting described above to minimize the residuals on η, we obtained corrected parameters of Δ*x* = 0.076 ± 0.012 nm, *k*_o_ = 5.1 × 10^–14^ ± 6.3 × 10^–14^ s^–1^. As shown in Figure 4d, without taking the biasing effect into account, the most probable unfolding force at each pulling speed was underestimated by ∼50 pN.

### Native Sacrificial Domains Are Optimal Shock Dissipators

**Figure 5:**
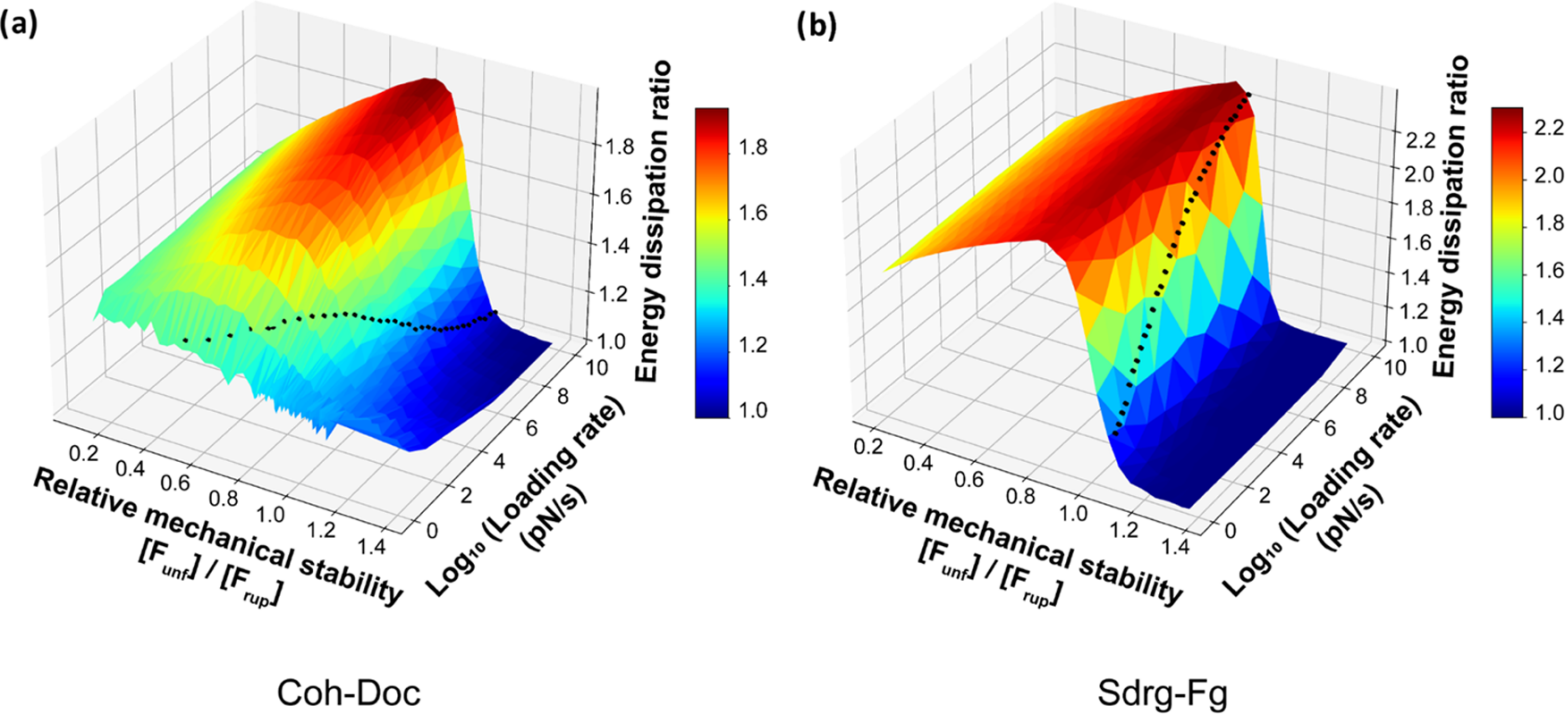
Monte Carlo simulations showing mechanical work (energy) dissipation as a function of the relative mechanical stability of the FP domain and the loading rate. Monte Carlo simulations under a constant loading rate were conducted on both (a) *C. perfringens* cohesin-dockerin RL system with variable stability of the FIVAR FP and (b) *S. epidermidis* SdrG:FgB RL system with variable stability of the B2 domain FP. The FP has a fixed initial off-rate *k*_o_ and the unfolding force distribution was tuned by adjusting both the energy barrier position (Δ*x*) and the loading rate from 1 to 10^10^ pN/s; 5000 simulations were performed for each energy barrier position and loading rate. The simulations corresponding to the WT sacrificial (a) FIVAR and (b) B2 are shown on the plots as black dots. Two-dimensional (2D) cutouts from these three-dimensional (3D) surface plots are shown in Supporting Information Figure 5.

To better understand how the mechanical stability of the sacrificial domain influences the total work required to dissociate the RL complex, we performed single-molecule Monte Carlo simulations^41−43^ on two systems: (1) *C. perfringens* cohesin-dockerin RL complex (Δ*x* = 0.77 nm, *k*_o_ = 0.011 s^–1^) with its adjacent FIVAR domain (Δ*x* = 0.594 nm, *k*_o_ = 6.2 × 10^–2^ s^–1^)^44^ (Figure 5a) and (2) *S. epidermidis* FgB:SdrG (Δ*x* = 0.063 nm, *k*_o_ = 1.8 × 10^–11^ s^–1^) with its adjacent B2 domain (Δ*x* = 0.076 nm, *k*_o_ = 5.1 × 10^–14^ s^–1^) (Figure 5b).

The external work required to dissociate the receptor–ligand complex during the pulling process for a given system was calculated as the average area under the force vs extension curve for an ensemble of simulated trajectories. We defined the work dissipation ratio as the ratio of the average work dissipated for a given RL system containing the FP to the average work dissipated for the same RL system lacking the FP. We calculated the work dissipation ratio for the WT adhesin systems (Figure 5, black dots) by simulating 5000 constant loading rate trajectories using numerical Monte Carlo with or without the respective FP domains. We next modulated the stability of the FP domain by changing the Δ*x* parameter, which changed the most probable unfolding force of the FP (Figure 5, [*F*_unf_]) away from that of the WT system. We simulated the pulling experiments with and without the altered FPs and calculated the work dissipation ratio while the FP stability was scanned from low to high. This was done for 25 different Δ*x* values for each FP/RL system under loading rate from 1 to 10^10^ pN/s (Figure 5).

As shown in Figure 5, with a fixed loading rate, a clear maximum in the work dissipation ratio was observed for both RL systems. Under different loading rates, the trend remains the same while the peak position shifts. For the *C. perfringens* Coh:Doc system (Figure 5a), maximum work dissipation was observed with [*F*_unf_]/[*F*_rup_] ranging from 0.62 to 0.98 and an eta value from 0.934 to 0.982. For the *S. epidermidis* FgB:SdrG system (Figure 5b), the maximum work dissipation was reached with [*F*_unf_]/[*F*_rup_] ranging from 0.81 to 0.89 and an eta value from 0.984 to 0.992. As the rate-dependent eta observations are caused by the competition between the rate-dependent dissipation of the FP and RL, the effect of enhanced work dissipation is also dependent on the loading rate. Two distinctive loading rate optima were found: the FIVAR sacrificial domain within the Coh:Doc system maximizes work dissipation at low loading rates, while for the Srdg-Fg system, the B domain maximizes work dissipation at a higher loading rate. For any adhesion system with a sacrificial domain, a loading rate or range of loading rates that correspond to the highest work dissipation could be found on the map, which represents the loading rate at which the dissipating effects of the sacrificial domain are maximized.

The Monte Carlo analysis (Figure 5) revealed that native FIVAR and B2 domains fused to their respective natural RL complexes (*C. perfringens* cohesin-dockerin and *S. epidermidis* SdrG:FgB, respectively) produced stretching ensembles with work dissipation ratios very close to the theoretical maximum values only within a given range of loading rates (FIVAR: ∼100 pN/s, B2 ∼10^10^ pN/s). This optimal value was achieved when the FP stability was situated slightly below the stability of the RL complex such that the ratio of most probable FP unfolding force to most probable RL complex rupture force ([*F*_unf_]/[*F*_rup_]) was near 0.8, corresponding to η ∼ 0.95. With the inclusion of the optimized sacrificial domain, the lifetime of the RL complexes can be enhanced under random fluctuating mechanical loads from the ambient environment (Supporting Information Figure 6). We note that at optimal values of η, the biasing effect is significant and the energy dissipation ratio is highest. To investigate the influence of linker length, we conducted numerical simulations also on systems with mock sacrificial domains that have the same contour length but lacked a folded structure (i.e., additional linker length) (Supporting Information Figures 2–4). Prior work has shown that the inclusion of compliant linkages can significantly influence the dynamic strength of biomolecular interactions in a loading-rate-dependent manner.^45,46^ The distribution of the simulated results is shown in Supporting Information Figures 3 and 4 for force ramp and constant pulling speed, respectively. We note that within a certain range of loading rates where the native FP shows the optimized energy dissipation, the inclusion of a stable mechanical fold could significantly improve the energy dissipation compared to an unfolded linker sequence; however, unstructured linkers performed as better dissipators than the folded dissipator within the regime where FP shows a low energy dissipation ratio (FIVAR in CohDoc: >10^2^ pN/s, B2 in SdrgFgB: <10^2^ pN/s).

## Discussion

When proteins are mechanically unfolded with an RL interaction as an anchor point, the competition between unbinding and unfolding pathways results in a biased distribution of unfolding forces for the FP. By quantifying η in large SMFS datasets, we were able to correct for this biasing effect and obtain corrected energy landscape parameter estimations for unfolding of several domains of interest (FIVAR, ddFLN4, B2). We demonstrated the concept on synthetic data as well as on an engineered system containing a VHH nanobody:mCherry interaction, where both unbiased and biased distributions were experimentally available. We then applied this algorithm to the FgB:SdrG-B2 system for which no sufficiently stable RL complex is available to obtain unbiased observations. Finally, we investigated the theoretically optimal η value that achieves maximal dissipation of mechanical work on average for an ensemble of pulling trajectories. This optimal η reflects a balance between strong sacrificial domains that dissipate large quantities of energy but do so infrequently, and weak sacrificial domains that dissipate small quantities of energy but do so with every loading event. Correction of the biasing effect results in changes to parameter estimates for both Δ*x* and *k*_off_. This can be understood because for a fixed set of RL parameters, Δ*x* of the FP influences the steepness of the loading rate dependency of FP unfolding, while the magnitude of the forces is dependent on both Δ*x* and *k*_off._ Both parameters can therefore influence the experimentally observed η values. Natural multidomain adhesive polyproteins must strike a balance that maximizes the average work dissipated by the given system to maintain adhesion. Our simulations also showed that high work dissipation for the polyprotein system can extend the bond lifetime under conditions of random velocity fluctuations and re-foldability of the FP. The fact that natural adhesin systems from *S. epidermidis* and *C. perfringens* produce polyproteins containing FPs and RLs with matched parameters near the optimum within a specific range of loading rates suggests a mechanical selection pressure could be at work in nature. In a similar way, other natural sacrificial domains found in bone and muscle could be further investigated under different physiological loading schemes for potential enhancement of work dissipation in tissues or synthetic materials in the future.

## Methods

### Gene Construction

The constructs for AFM measurements were (i) SdrG-ddFLN4-ELP-His-ybbR; (ii) Fgß-FIVAR-ELP-His-ybbR; (iii) SdrG-B2-ELP-His-ybbR; (iv) Fgß-Coh7-ddFLN4-ELP-His-ybbR; (v) VHH-ddFLN4-His-ybbR; and (vi) mCherry-FIVAR-His-ybbR. The plasmid pET28a_SdrG-B1-B2-HIS-HRV3C-ybbr and pET28a_ybbr-HIS-ddFLN4-8GS-FIVAR-Doc were kind gifts from Hermann Gaub’s lab at Ludwig-Maximilians-Universität Munich. The Staphylococcus epidermidis SdrG N2 and N3 domain genes, the Staphylococcus epidermidis SdrG B1 domain, ddFLN4 domain (*D. discoideum* 4th filamin), FIVAR domain from C. perfringens, and Coh7 domain (seventh cohesin domain of CipA from Clostridium thermocellum) were inserted into a pET28a vector containing ELP-HIS-ybbr using Gibson assembly, respectively. Construction of (v) and (vi) was introduced in a previous publication.^34^ Final open reading frames of all constructs were confirmed by Sanger sequencing (Microsynth AG). The Addgene information of the plasmids (i to vi) and the complete sequences of all protein constructs used are listed in the Supporting Information.

### Protein Expression and Purification

All proteins were expressed in *E. coli* BL21(DE3). Precultures of 5 mL in LB medium containing 50 μg/mL Kanamycin, grown overnight at 37 °C, were inoculated in 200 mL of ZYM-5052 autoinduction media^47^ containing Kanamycin and grown for 10 h at 37 °C. Bacteria were harvested by centrifugation at 4000*g*, and pellets were stored at −80 °C until purification. The cell pellet was resuspended in Lysis Buffer (50 mM TRIS, 50 mM NaCl, 5 mM MgCl_2_, 0.1% (v/v) TritonX-100, pH 8.0) including 100 μg/mL Lysozyme and lysed by sonication, followed by centrifugation at 16,000*g* for 30 min at 4 °C. The His6-tagged proteins were purified using a His-Trap FF column, followed by desalting using a His-Trap Desalting column on AKTA Pure system followed by size exclusion. Protein concentrations were determined by absorbance at 280 nm.

### Single-Molecule Force Spectroscopy on AFM

Immobilization of the fusion protein on the AFM cantilever as well as the glass surface was achieved as introduced in previous papers.^3,34,48^ In brief, Sulfo-SMCC was covalently bonded to the silanized surface of the AFM cantilever and the glass surface for constructs containing ELP included constructs, and a 5k Mal-PEG-NHS linker was conjugated for non-ELP constructs, followed by conjugation of CoA on the surface. Then, the ybbR labeled fusion proteins were conjugated to CoA using an SFP reaction. All measurements were conducted in TBS buffer (25 mM Tris, 75 mM NaCl, pH 7.4) under a constant pulling speed.

### Monte Carlo Simulation of Forced Pulling Process

To validate the framework of biasing effect between the receptor–ligand dissociation and FP domain unfolding, a Monte Carlo simulation based on Kramers theory was used to simulate the forced pulling process under a constant speed or a constant force loading rate protocol. First, a series of evenly distributed extension values for the molecular system *X*(*t*) was generated, by which the applied force *F*(*t*) could be calculated using a worm-like chain (WLC) model (eq 7) with a persistence length of 0.365 nm.
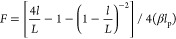
7

For the constant pulling speed mode, to simulate the force spectroscopy on AFM, bending of the AFM cantilever was added to the molecular extension to give the AFM head height *H*(*t*) (eq 8) using a spring constant of ν equal to 90 pN/nm (AC40 BioLever mini cantilever, Bruker) and the time series could be generated by applying a constant pulling speed V on the AFM head height
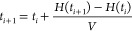
8

For a constant force loading rate mode (Force ramp) where the pulling force is applied using a constant loading rate LR with respect to time following: *F*_*ti*_*– F*_*ti*–1_ = LR*(*t*_*i*_*– t*_*i*–1_), the time series could be generated with applied force loading rate LR
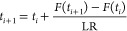
9

Then, the simulation of the dissociation process for FP unfolding and RL rupture during the forced pull test was conducted within each time interval along the time axis until the RL complex ruptured. To obtain the probabilities for the receptor–ligand dissociation and FP domain unfolding within each timestep, the force-dependent off-rate *k*(*F*) was integrated over each time interval (*t_i_*, *t*_*i*+1_)
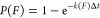
10where *k*(*F*) can be drawn from eq 11 following the Bell–Evans model or from eq 12 following the Dudko–Hummer–Szabo model as an alternative
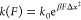
11
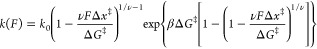
12where *β*^–1^ = *k*_B_*T*. To test if the dissociation happens for the FP unfolding/RL complex rupture, the dissociation probability is compared to a random number between zero and unity to test if the rupture or unfolding event occurs within each time interval. When the FP domain unfolding event occurs, a released contour length is added to the total molecular length while both force and extension series are updated correspondingly. The extension of the released contour length after the unfolding events was simulated following Hooke’s law with a modulus at 90 pN/nm, which is the stiffness of the AFM cantilever. For each system, 1000 curves were generated. The aforementioned Monte Carlo simulations were performed using Python, the code and corresponding instructions of which are available at https://github.com/Nash-Lab/Monte-Carlo-Methods.

### Simulating Eta Observation under Constant-Speed Pulling Experiment using Monte Carlo Simulation

To correct energy landscape parameters by minimizing η residuals, a numerical calculation was conducted to yield the rate-dependent η following eqs 4 and 5 for the nonlinear fitting process. For the constant-speed loading protocol, η at a given set of energy barrier parameters was given by Monte Carlo simulation of constant-speed pulling test. To ensure the convergence of the nonlinear fitting process, 10,000 force extension curves were generated using the simulation introduced above and the eta value was calculated by the ratio of the force curves that show FP domain unfolding. Since the experimental results fitted in the paper were collected using a constant pulling speed protocol, the η observations could be more precisely described using this approach with Monte Carlo simulation. We note that the differences between the fitted energy landscape parameters obtained from the numerical integration approach and from direct Monte Carlo simulation of the system are negligible. We suggest that to correct the energy profile from biased observations, numerical calculations following eqs 4 and 5 should be used under an approximation of constant loading rate to avoid long Monte Carlo simulation run times.

## Supporting Information Available

Addgene plasmid information, protein amino acid sequences, and additional Monte Carlo simulations (PDF)
