# Supplementary material for: Optimal Sacrificial Domains in Mechanical Polyproteins: S. epidermidis Adhesins Are Tuned for Work Dissipation
Source: JACS Au. 2022 May 18;2(6):1417–27. doi: 10.1021/jacsau.2c00121 (PMC9241160; doi:10.1021/jacsau.2c00121)
Supplement: Supplementary file 1 — au2c00121_si_001.pdf [file au2c00121_si_001.pdf]

Supporting Information

Optimal Sacrificial Domains in Mechanical Polyproteins: *S. epidermidis* Adhesins are Tuned for Work Dissipation

*Haipei Liu[1,2], Zhaowei Liu[1,2], Byeongseon Yang[1,2], Joanan Lopez Morales[1,2], and Michael A. Nash[1,2 *]*

[1] Department of Chemistry, University of Basel, 4058 Basel, Switzerland

[2] Department of Biosystems Science and Engineering, ETH Zurich, 4058 Basel, Switzerland

* Correspondence to: michael.nash@unibas.ch

Plasmids and protein sequences used in AFM-SMFS

Plasmids available on addgene:

Addgene #168047: SdrG-ddFLN4-ELP-HIS-ybbR

Addgene #169134: SdrG-B2-ELP-HIS-ybbR

Addgene #169135: Fgß-Coh7-ddFLN4-ELP-HIS-ybbR

Addgene #169136: Fgß-FIVAR-ELP-HIS-ybbR

Addgene #169137: VHH-ddFLN4-HIS-ybbR

Addgene #169138: mCherry-FIVAR-HIS-ybbR

Protein sequences for AFM-SMFS including marker domains:

SdrG-ddFLN4-ELP-HIS-ybbR

(Addgene accession code 168047)

MGTEQGSNVNHLIKVTDQSITEGYDDSDGIIKAHDAENLIYDVTFEVDDKVKSGDTMTVNIDKNTVPSDLTDSFAIPKIK
DNSGEIIATGTYDNTNKQITYTFTDYVDKYENIKAHLKLTSYIDKSKVPNNNTKLDVEYKTALSSVNKTITVEYQKPNEN
RTANLQSMFTNIDTKNHTVEQTIYINPLRYSAKETNVNISGNGDEGSTIIDDSTIIKVYKVGDNQNLPDSNRIYDYSEYED
VTNDDYAQLGNNNDVNINFGNIDSPYIIKVISKYDPNKDDYTTIQQTVTMQTTINEYTGEFRTASYDNTIAFSTSSGQGQ
GDLPPEGSGSGSGSADPEKSYAEGPGLDGGECFQPSKFKIHAVDPDGVHRTDGGDGFVVTIEGPAPVDPVMVDNGDGT
YDVEFEPKEAGDYVINLTLDGDNVNGFPKTVTVKPAPGSGSGSHGVGVPGMGVPGVGVPGVGVPGVGVPGVGVPGV
GVPGVGVPGVGVPGEGVPGEGVPGVGVPGMGVPGVGVPGVGVPGVGVPGVGVPGVGVPGVGVPGVGVPGEGVPGE

GVPGVGVPGMGVPGVGVPGVGVPGVGVPGVGVPGVGVPGVGVPGVGVPGEGVPGEGVPGWRGHHHHHHGSDSLEFI
ASKLA

SdrG-B2-ELP-HIS-ybbR

(Addgene accession code 169134)

MGTEQGSNVNHLIKVTDQSITEGYDDSDGIIKAHDAENLIYDVTFEVDDKVKSGDTMTVNIDKNTVPSDLTDSFAIPKIK
DNSGEIIATGTYDNTNKQITYTFTDYVDKYENIKAHLKLTSYIDKSKVPNNNTKLDVEYKTALSSVNKTITVEYQKPNEN
RTANLQSMFTNIDTKNHTVEQTIYINPLRYSAKETNVNISGNGDEGSTIIDDSTIIKVYKVGDNQNLPDSNRIYDYSEYED
VTNDDYAQLGNNNDVNINFGNIDSPYIIKVISKYDPNKDDYTTIQQTVTMQTTINEYTGEFRTASYDNTIAFSTSSGQGQ
GDLPPEKYSLGNYVWYDTNKDGIQGDDEKGISGVKVTLKDENGNIISTTTTDENGKYQFDNLNSGNYIVHFDKPSGMT
QTTTDSGDDDEQDADGEEVHVTITDHDDFSIDNGYYDDDSGSGSHGVGVPGMGVPGVGVPGVGVPGVGVPGVGVPG
VGVPGVGVPGVGVPGEGVPGEGVPGVGVPGMGVPGVGVPGVGVPGVGVPGVGVPGVGVPGVGVPGVGVPGEGVPG
EGVPGVGVPGMGVPGVGVPGVGVPGVGVPGVGVPGVGVPGVGVPGVGVPGVGVPGEGVPGEGVPGWRGHHHHHHGSDSLE
FIASKLA

Fgß-Coh7-ddFLN4-ELP-HIS-ybbR

(Addgene accession code 169135)

NEEGFFSARGHRPLDGSGSGSGSAVRIKVDTVNAKPGDTVRIPVRFSGIPSKGIANCDFVYSYDPNVLEIIEIEPGELIVDP
NPTKSFDTAVYPDRKMIVFLFAEDSGTGAYAITEDGVFATIVAKVKSGAPNGLSVIKFVEVGGFANNDLVEQKTQFFDG
GVNVGGSGSGSGSADPEKSYAEGPGLDGGECFQPSKFKIHAVDPDGVHRTDGGDGFVVTIEGPAPVDPVMVDNGDGT
YDVEFEPKEAGDYVINLTLDGDNVNGFPKTVTVKPAPGSGSGSHGVGVPGMGVPGVGVPGVGVPGVGVPGVGVPGV
GVPGVGVPGVGVPGEGVPGEGVPGVGVPGMGVPGVGVPGVGVPGVGVPGVGVPGVGVPGVGVPGVGVPGEGVPGE
GVPGVGVPGMGVPGVGVPGVGVPGVGVPGVGVPGVGVPGVGVPGVGVPGEGVPGEGVPGWRGHHHHHHGSDSLEFI
ASKLA

Fgß-FIVAR-ELP-HIS-ybbR

(Addgene accession code 169136)

NEEGFFSARGHRPLDGSGSGSGSDKTNLGELINQGKSLLDESVEGFNVGEYHKGAKDGLTVEINKAEEVFNKEDATEEE
INLAKESLEGAIARFNSLLIEESTGSGSGSHGVGVPGMGVPGVGVPGVGVPGVGVPGVGVPGVGVPGVGVPGVGVPGE
GVPGEGVPGVGVPGMGVPGVGVPGVGVPGVGVPGVGVPGVGVPGVGVPGVGVPGEGVPGEGVPGVGVPGMGVPGV
GVPGVGVPGVGVPGVGVPGVGVPGVGVPGVGVPGEGVPGEGVPGWRGHHHHHHGSDSLEFIASKLA

VHH-ddFLN4-HIS-ybbR

(Addgene accession code 169137)

MAQVQLVESGGSLVQPGGSLRLSCAASGRFAESSSMGWFRQAPGKEREFVAAISWSGGATNYADSAKGRFTLSRDNT
KNTVYLQMNSLKPDDTAVYYCAANLGNYISSNQRLYGYWGQGTQVTVSSPFTGSGSGSGSAGTGSGADPEKSYAEGP
GLDGGESFQPSKFKIHAVDPDGVHRTDGGDGFVVTIEGPAPVDPVMVDNGDGTYDVEFEPKEAGDYVINLTLDGDNVN
GFPKTVTVKPAPSGHHHHHHGSDSLEFIASKLA

mCherry-FIVAR-HIS-ybbR

(Addgene accession code 169138)

MVSKGEEDNMAIIKEFMRFKVHMEGSVNGHEFEIEGEGEGRPYEGTQTAKLKVTKGGPLPFAWDILSPQFMYGSKAYV
KHPADIPDYLKLSFPEGFKWERVMNFEDGGVVTVTQDSSLQDGEFIYKVKLRGTNFPSDGPVMQKKTMGWEASSERM
YPEDGALKGEIKQRLKLKDGGHYDAEVKTTYKAKKPVQLPGAYNVNIKLDITSHNEDYTIVEQYERAEGRHSTGGMDE
LYKGSGSGSGSDKTNLGELINQGKSLLDESVEGFNVGEYHKGAKDGLTVEINKAEEVFNKEDATEEEINLAKESLEGAI
ARFNSLLIEESTGSVVPGWLHHHHHHGSDSLEFIASKLA

Supplementary Figures

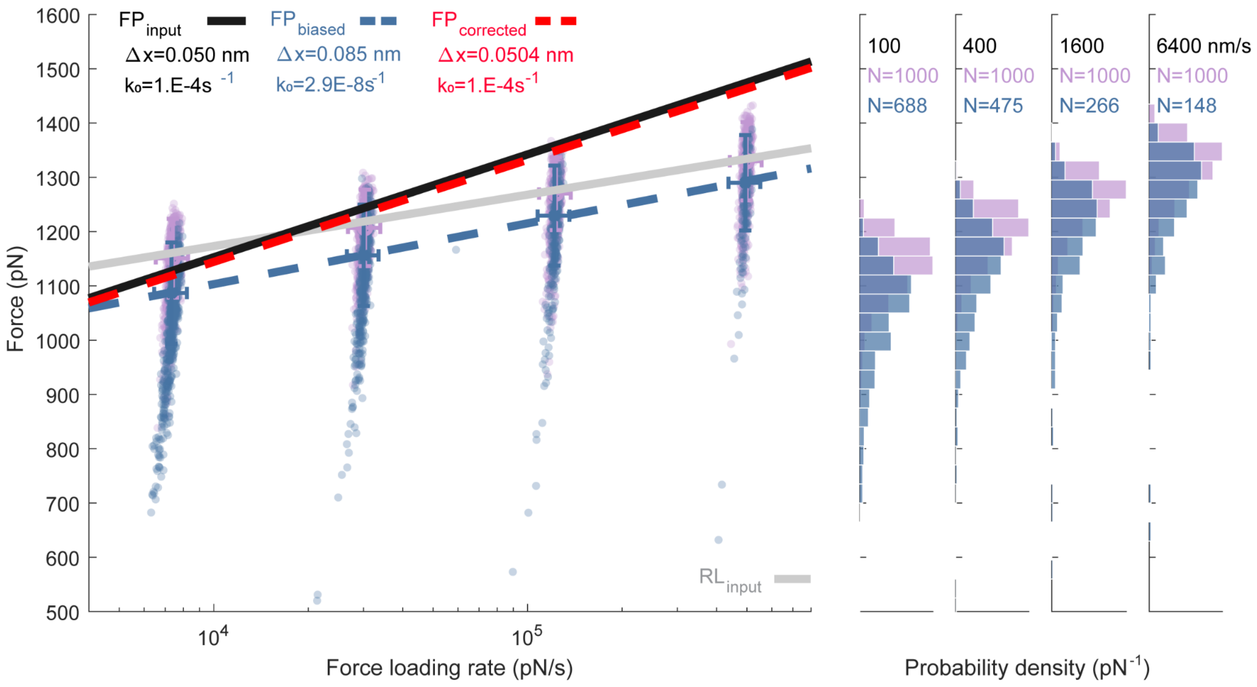

Supplementary Figure 1. Validation of the correction algorithm on a synthetic SMFS data set generated using Monte Carlo simulation. The forced pulling of the receptor-ligand complex (RL) with a fingerprint domain (FP) was simulated with constant pulling speeds of 100, 400, 1600, and 6400 nm/s using a Monte Carlo method. At each pulling speed, 1000 force extension curves were generated. The histograms on the right show the force values of RL rupture events (purple) and FP unfolding events (blue) obtained in the simulation. Load was applied using a cantilever with a spring constant of 90 pN/nm. The RL complex was simulated using Bell-Evans parameters of Δx=0.1 nm, k_o=1.0 × 10^{-10} s^{-1} (gray solid line) as input to the simulation. The FP unfolding was simulated using the parameters Δx=0.05 nm, k_o=1.0 × 10^{-4} s^{-1} as input (black solid line). A biased energy profile of Δx=0.085 nm, k_o=2.9 × 10^{-8} s^{-1} was obtained from fitting the FP unfolding with the Bell-Evans model (shown in blue dashed line). It was then corrected using the Monte Carlo simulation approach based on the eta observation and the unbiased RL energy profile to yield the unbiased (i.e., corrected) energy profile of Δx=0.0504 nm, k_o=1.0 × 10^{-4} s^{-1} (shown in red dashed line). This shows that the correction algorithm based on minimizing residuals on eta can recover the true input parameters for the FP.

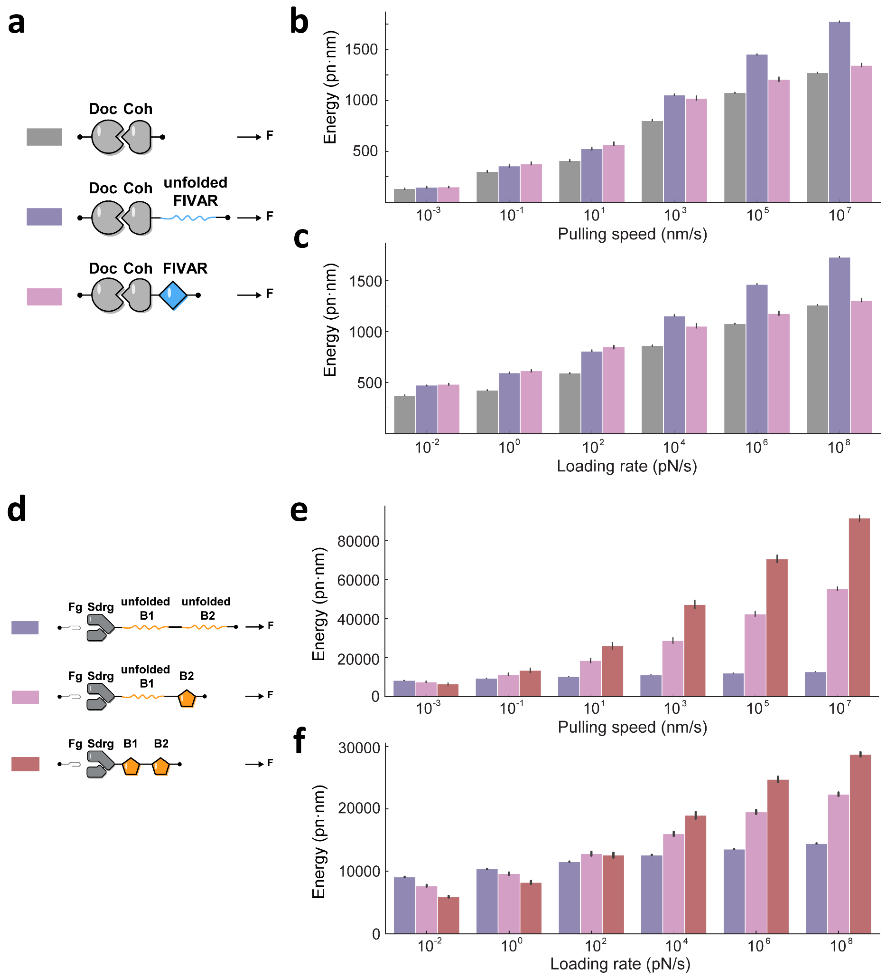

Supplementary Figure 2. Work dissipation by an RL-linked polyprotein under mechanical load. The forced pulling process is simulated using Monte Carlo methods on receptor-ligand systems: (a, b, c) *C. perfringens* Coh:Doc interaction with FIVAR as the FP domain. (d, e, f) *S. epidermidis* SdrG:FgB interaction with B1 and B2 domains as FPs. Polyproteins were loaded using two loading schemes: (b and e) constant speed Monte Carlo at pulling speeds from 10^{-3} to 10^{7} nm/s , and (c and f) force ramp Monte Carlo with a constant loading rate from 10^{-2} to 10^{8} pN/s. For each loading rate, 1000 force extension traces were simulated. The distributions of the external work required to break the RL interaction are given in **Supplementary Figure 3** and **Supplementary Figure 4**. Here we tested the addition of unstructured linkers length and find they are capable of dissipating more work than folded FPs at low loading rates for SdrG:FgB at low loading rates, while for Coh:Doc the unstructured linkers show better performance at high loading rates, where the biasing effect works in such a way that in the majority of trajectories, the FP remained folded. In this case the presence of the folded FP served to lower the work required to stretch the systems and rupture the RL as compared to the unfolded flexible linkers.

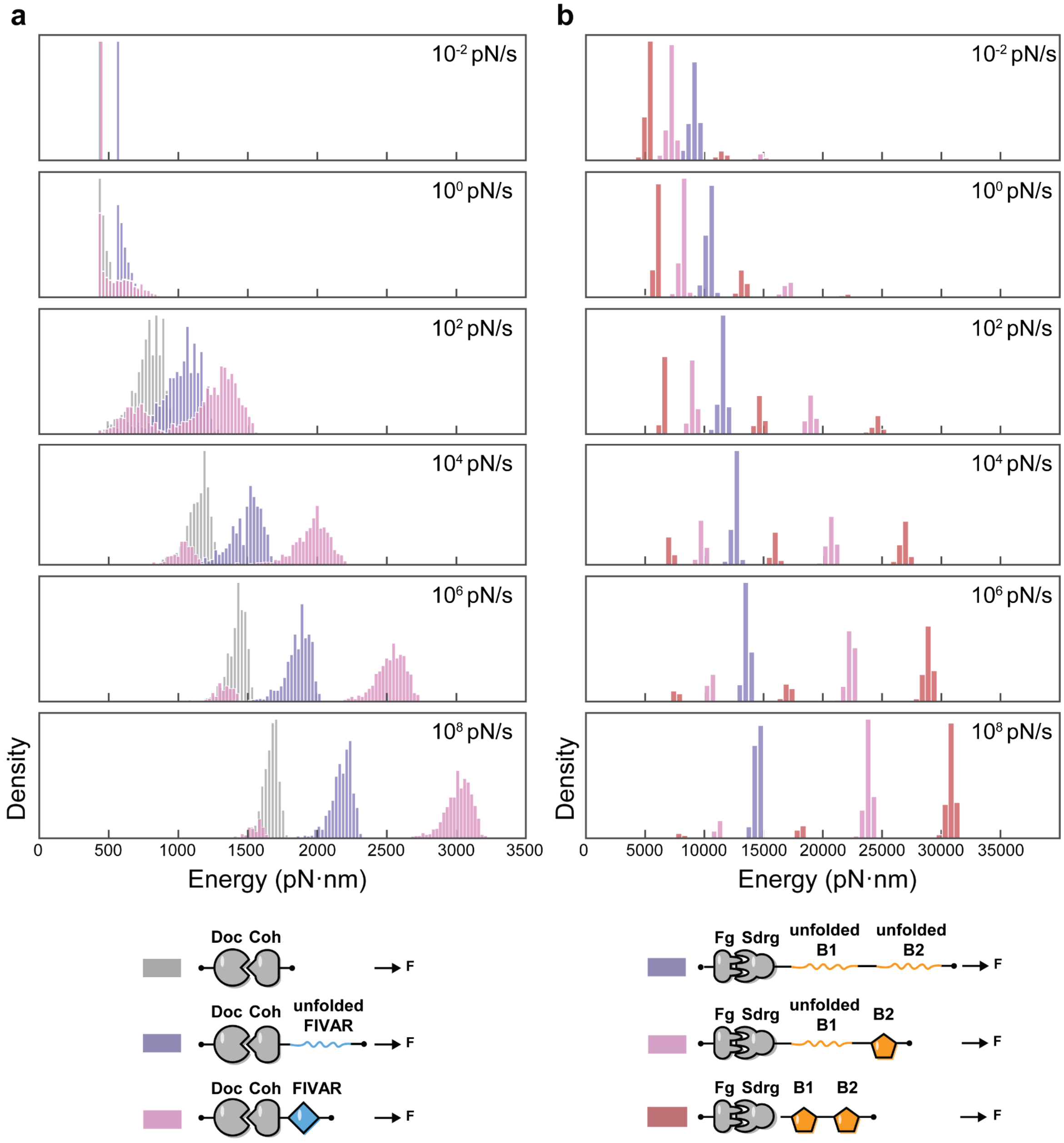

Supplementary Figure 3. Work required to dissociate RL-linked polyproteins systems with an energy dissipator FP domain under force ramp mode. The forced pulling process was simulated using the Monte Carlo method for the two given RL systems and respective FPs: (a) SdrG-Fg RL interaction along with B domains from *S. epidermidis* and (b) cohesin-dockerin interaction along with FIVAR domain from *C. perfringens*. Systems were loaded using force ramp mode with a constant loading rate from 10^{-2} to 10^{8} pN/s. For each loading rate, 1,000 force extension traces were simulated.

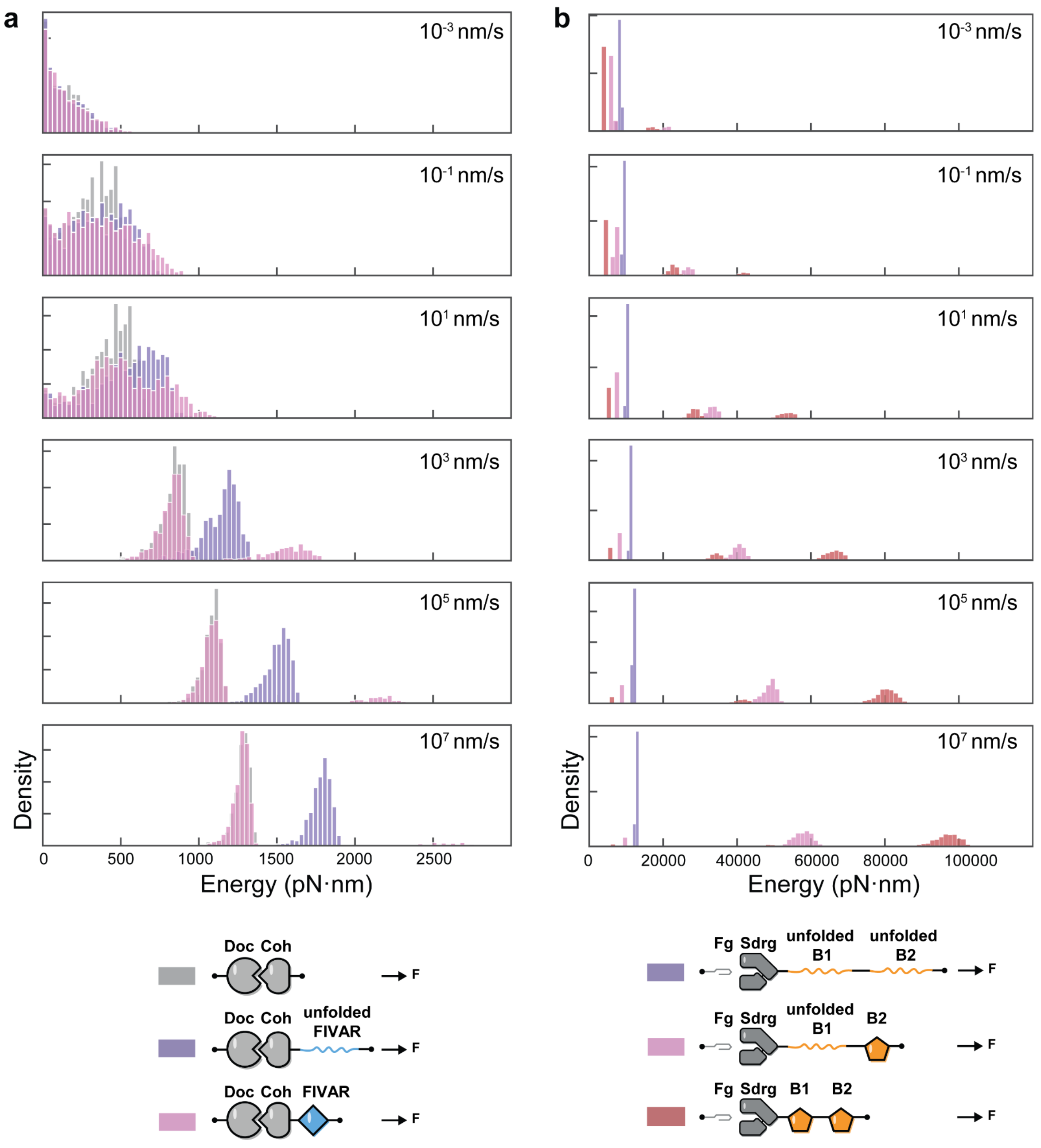

Supplementary Figure 4. Work required to dissociate RL-linked polyproteins systems with an energy dissipator FP domain under constant speed mode. The constant speed pulling process was simulated using Monte Carlo methods on receptor-ligand systems: (a) SdrG-Fg interaction along with B domains from *S. epidermidis* and (b) cohesin-dockerin interaction along with FIVAR domain from *C. perfringens*. Systems were loaded in constant speed mode with pulling speeds from 10^{-3} to 10^{7} nm/s. For each speed, 1,000 force extension traces were simulated.

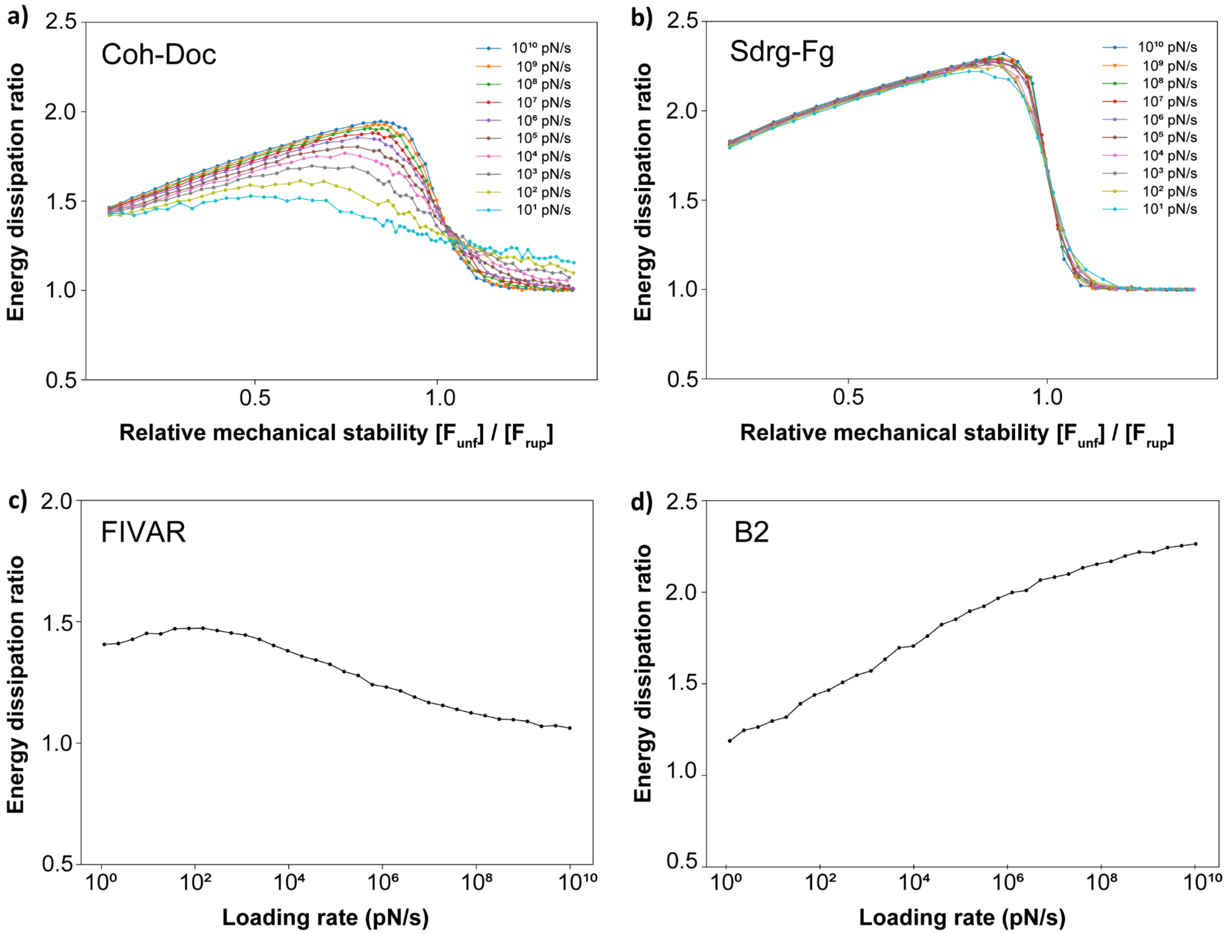

Supplementary Figure 5. Monte Carlo simulations showing the energy dissipation ratio, representing the amount of external work required to stretch and dissociate the polyprotein system normalized to the same quantity for the RL system lacking the FP. (a) C. perfringens cohesin-dockerin RL system with variable stability of the FP domain achieved by modulating Δx only and fixing k_{off} to that of the WT FIVAR FP. (b) *S. epidermidis* SdrG:FgB RL system with variable stability of the FP domain achieved by modulating Δx only and fixing k_{off} to that of the WT B2 FP. The loading rate dependency of the energy dissipation ratio shown for polyprotein systems containing native RL and sacrificial FP domains (c) Coh-Doc/FIVAR and (d) SdrG-FgB/B2.

a

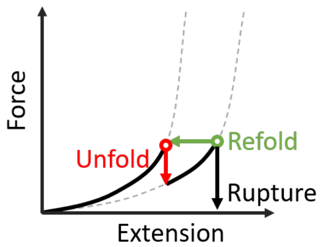

b

		w/o refolding		refolding Enabled	
Shock dissipator	-	B2	B1, B2	B2	B1, B2
Lifetime [s]	0.12 ± 0.07	0.29 ± 0.15	0.69 ± 0. 54	0.50 ± 0.42	1.22 ± 1.14

c

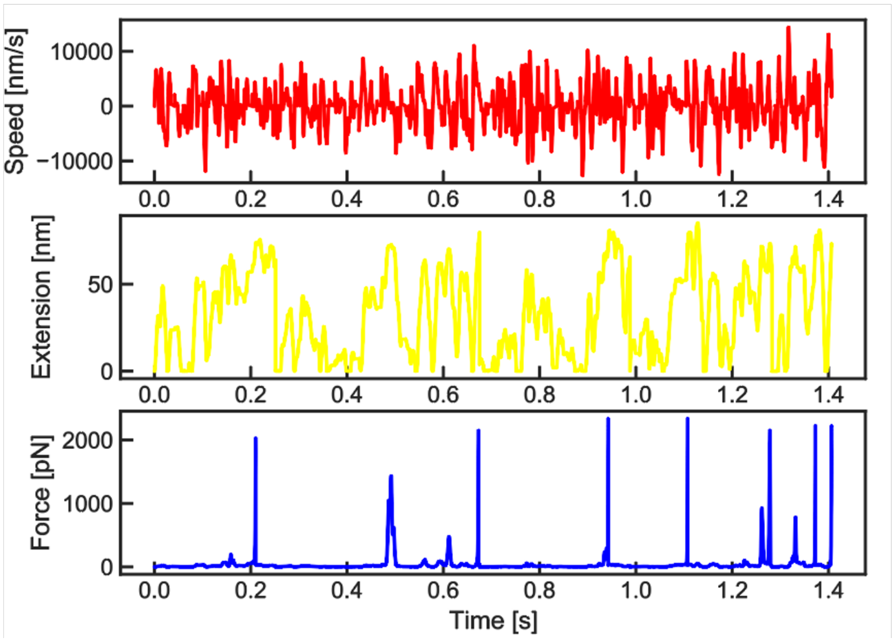

Supplementary Figure 6. Monte Carlo simulations showing the lifetime of the SdrG-FgB polyprotein system under random velocity noise. A random pulling speed from the Perlin noise function with an amplitude at 10000 nm/s was applied as input to a Monte Carlo protocol. The rupture of the SdrG-FgB complex was simulated using energy profiles: Δx=0.063 nm, k_o=1.8 × 10^{-11} s^{-1}. The unfolding of the shock dissipator domain was simulated using energy profiles of B1: Δx=0.083 nm, k_o=5.4 × 10^{-15} s^{-1} and B2: Δx=0.076 nm, k_o=5.1 × 10^{-14} s^{-1}. To simulate the refolding process of the shock dissipator, an approximation of energy profiles (B1: Δx=0.083 nm, k_o=10 s^{-1}, B2: Δx=0.076 nm, k_o=10 s^{-1}) was used. For each group, 1,000 force-time traces were simulated. (b) The total lifetime of the polyprotein system could be significantly increased by the inclusion of the shock dissipator domain. (c) Typical simulation record with multiple refolding events after the shock dissipator was unfolded by the mechanical load, by which the lifetime could be enhanced.
